# Selection of Representative Questionnaire Items from the Asthma Control Test

**DOI:** 10.3390/jpm12111913

**Published:** 2022-11-16

**Authors:** Min-Seok Chang, Iseul Yu, Sunmin Park, Ji-Ho Lee, Seok Jeong Lee, Won-Yeon Lee, Suk Joong Yong, Meounggun Jo, Sang-Ha Kim

**Affiliations:** 1 Department of Internal Medicine, Yonsei University Wonju College of Medicine, Wonju 26426, Republic of Korea; 2 Department of Innovation and Convergence, Hoseo University, Ansan 31499, Republic of Korea

**Keywords:** asthma, questionnaire, representative questionnaire, digital healthcare

## Abstract

Asthma is a disease characterized by the appearance of transient or persistent symptoms in response to allergens, viral upper respiratory infections, and cold air. Asthma treatment aims to control, rather than cure, and digital systems can be useful in this regard. However, conventional assessment methods for asthma control are not suitable for digital healthcare. Therefore, we aimed to select representative questionnaire items suitable for digitally assessing the asthma control status. We analyzed the Asthma Control Test (ACT) and selected representative items. Throughout the year 2020, ACT results (2019 in total) collected from patients (>18 years old) with a principal diagnosis of asthma were analyzed. Individual questionnaire items were tested using Pearson’s correlation and receiver operating characteristic curves. Of the five questionnaire items, Q1, Q2, Q3, and Q5 yielded significant findings. Among these questionnaires, Q2 was the most descriptive and correlated questionnaire. Q5 was also significant but it was excluded since it was unable to apply to the digital health care system for asthma assessment method. The remaining three questionnaire items were selected and their sensitivity and specificity were assessed. Eight methods were analyzed, and the sum of scores of Q1–Q3 had the highest sensitivity and specificity (97% and 91%, respectively). The results suggested that, instead of the full items of ACT, the sum of Q1–Q3 can be used to assess the asthma control status. These findings will serve as the foundation for developing digital asthma control assessment tools.

## 1. Introduction

Asthma is a disease in which symptoms, such as wheezing, breathing difficulties, and cough, are caused by exposure to allergens, viral upper respiratory infections, exercise, and cold air [1]. The symptoms are either transient or persistent. Treatment for asthma is aimed at maintaining the disease under control and preventing acute exacerbation rather than curing the disease. Therefore, early intervention is essential if there changes in asthma status [2].

Digital healthcare can facilitate such asthma control assessments and offers the advantage of allowing real-time access to data, which can be helpful considering the goal of asthma management [3]. In this regard, some studies have attempted to use digital healthcare as the basis for an asthma control assessment tool, and although limited, some benefits have been demonstrated [4,5,6]. However, the assessment standards for the level of symptom control used in these studies were mostly the same as those used in clinical trials [7,8]. Other studies used their own survey methods, without existing validation, for evaluating the level of asthma symptom control [9,10].

Among the traditional asthma assessment tools [11,12], the Asthma Control Test (ACT) consists of five questionnaire items that are completed when visiting a hospital, and this tool is used for assessing asthma control status by summing the scores of each questionnaire item [13,14]. However, the ACT should be able to be filled out on the scheduled or unscheduled hospital visits. Consequently, it is not suitable for digital healthcare systems that can record the immediate control status. Therefore, this study aimed to identify the ACT questionnaire items that are applicable for a digital healthcare system and select representative questionnaire items to provide evidence for future use.

## 2. Materials and Methods

### 2.1. Participants of the Study

**Figure 1:**
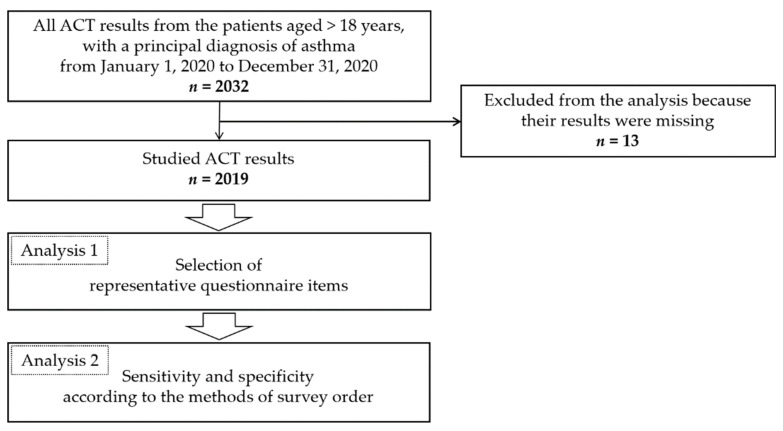
In the ACT-based evaluation, the analysis was divided into “Analysis 1” for selecting representative questionnaire items and “Analysis 2” to determine the combination for investigation.

This study involved patients aged >18 years who visited Yonsei University Wonju Severance Christian Hospital, Gangwon-do, Republic of Korea with a principal diagnosis of asthma during the one-year period from 1 January 2020 to 31 December 2020. All ACT results obtained from the target patients were collected. Thus, a total of 2032 ACT assessments were identified from 682 asthmatic patients. Some ACT results were made by the same patient because they visited the hospital more than once a year. Thirteen of the ACT results were excluded from the analysis because their results were missing. The remaining 2019 ACT assessments were examined (Figure 1). The sex and age data of the 2019 ACT results are shown in Table 1.

This study was conducted after undergoing a review and obtaining approval (CR321105) from the Institutional Review Board of Yonsei University Wonju Severance Christian Hospital. The requirement of informed patient consent was waived due to the retrospective design of the study.

### 2.2. Methods

#### 2.2.1. ACT

The ACT, developed by Nathan et al., is a well-known questionnaire for evaluating asthma control status. In the previous study, at the time the questionnaire was developed, patients arriving at the hospital answered 22 questions selected by experts, and the final five items were selected [13]. It is known that the ACT score reflects the degree of change in the forced expiratory volume in 1 s and asthma symptoms [13,14]. The current version includes the final five items selected through forward stepwise logistic regression analyses in previous research [13]. The ACT questionnaire items are as follows: In the past 4 weeks, Q1: How much of the time did your asthma keep you from getting as much done at work, school, or home? Q2: During the past 4 weeks, how often have you experienced shortness of breath? Q3: During the past 4 weeks, how often did your asthma symptoms wake you up at night or earlier than usual in the morning? Q4: During the past 4 weeks, how often have you used rescue medication? Q5: How would you rate your asthma control over the past 4 weeks?

All questionnaire items are rated on a 5-point scale, with higher scores reflecting greater asthma control. The ACT total score is calculated by summing the scores obtained for each of the items, and the asthma control status is evaluated according to the total score. An ACT total score of 5–15 indicates very poorly controlled asthma, scores of 16–19 indicate not well-controlled asthma, and a score of 20–25 indicates well-controlled asthma [13,15]. Since the one of object of this study is develop to develop representative questionnaire items for making a modified new tool for asthma assessment using a digital healthcare system, which is designed to find a change of asthma status early but not designed to find how severe asthma is, this study divided patients into two groups. Not well-controlled and poorly controlled are the same in the design of the new assessment tool we are designing [11]: Group 1 included patients with an ACT total score of 19 or less, indicating not well-control of asthma, while Group 2 included patients with a score of 20 or higher, indicating well-controlled asthma.

#### 2.2.2. Selection of Representative Questionnaire Items (Analysis 1)

This analysis was conducted to select representative questionnaire items suitable for use with digital healthcare systems. The correlation with the ACT total score was analyzed for each item in order to confirm which item can distinguish between Groups 1 and 2.

#### 2.2.3. Sensitivity and Specificity According to the Methods of Survey Order Using the Selected Representative Questionnaire Items (Analysis 2)

The discernment of the two groups divided by the ACT total score was expected to differ depending on the order of survey for the selected questionnaire items. The questionnaire item with the highest explanatory power and relevance was included in the survey order first, and then all possible survey order methods were considered, and the sensitivity and specificity of each method were evaluated.

#### 2.2.4. Statistical Analysis

To determine which questionnaire items could distinguish Groups 1 and 2, the correlation between the scores for each questionnaire items and the ACT total score was analyzed using Pearson correlation coefficients. For this analysis, the correlation between the scores for each questionnaire item and the ACT total score including this item was first obtained. In this case, since this correlation was influenced by multicollinearity, an additional correlation analysis was conducted to obtain the individual relationship between the scores for each questionnaire item and the total score after excluding that item. In addition, the degree of separation between Groups 1 and 2 was compared using receiver operating characteristic (ROC) curve analysis.

IBM SPSS v23.0 and Microsoft Office Excel 2016 were used for statistical analysis. Statistical significance was set at *p* < 0.05.

## 3. Results

### 3.1. Selection of Representative Questionnaire Items

All five ACT questionnaire items showed correlations with the total ACT score (Table 2).

The scores for Q1, Q2, Q3, and Q5 showed greater correlations with the total ACT score than the score for Q4. Additionally, the correlation between the score for each questionnaire item and the total score after excluding the score for that item was calculated, and the results are provided in Table 3.

Similar to the previous analysis, the scores for Q1, Q2, Q3, and Q5 showed greater correlation with the total score than the score for Q4.

**Figure 2:**
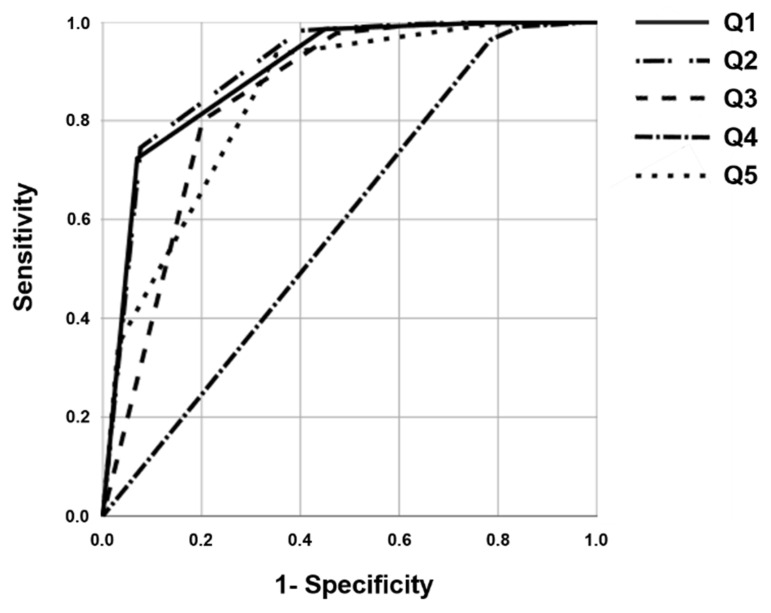
Receiver operating characteristic (ROC) curve to determine how well the scores for each ACT questionnaire item distinguished between groups (Group 1) in which asthma symptoms were not well controlled.

The ROC curve was used to determine how well the ACT questionnaire item scores explained the results. The explanatory power of each questionnaire item score was compared using the area under the curve. The results are presented in Figure 2 and Table 4.

The results of the ROC curve analysis confirmed that the AUC values of Q1, Q2, Q3, and Q5 were greater than that of Q4.

### 3.2. Sensitivity and Specificity According to the Methods of Survey Order Using the Selected Representative Questionnaire Items

Even though questionnaire item 5 (How would you rate your asthma control during the past 4 weeks?) showed high AUC than Q4, this item was about the overall evaluation of asthma control status, and it was excluded from the selection because of the difficulty in using this item for survey with a digital healthcare system. Thus, the questionnaire items suitable for diagnosing the asthma symptom control status according to the ACT total score were Q1, Q2, and Q3. It was analyzed using only these two to three questionnaires whether to seek if it still has enough power to represent ACT or not. Various survey order methods that can be combined using these items were identified. First of all, Q2, which had the highest explanatory power and relevance among these items, was included in the questionnaire in the first order, and then it was confirmed which method would be better to use, Q1 or Q3, which had the next highest explanatory power and relevance. The sensitivity and specificity for diagnosing Group 1 whose asthma symptoms were not well-controlled according to each survey order method were confirmed.

The number of survey order methods was divided into eight categories.

(1) Only examine Q2 to check for abnormal findings,
(2) Examine Q2 and Q1 to check for at least one abnormal finding,
(3) Examine Q2 and Q1 to check for both abnormal findings,
(4) Examine Q2, Q1, and Q3 to check for at least one abnormal finding,
(5) Examine Q2, Q1, and Q3 to check for at least two abnormal findings,
(6) Examine Q2, Q1, and Q3 to check for all three abnormal findings,
(7) Examine Q2 and one questionnaire item out of Q1 and Q3 to calculate the sum of the two (7-1: Questionnaire items 2 and 1, 7-2: Questionnaire items 2 and 3).
(8) Examine Q2, Q1, and Q3 to calculate the sum of all three.

For survey order methods (1) through (6), when the score for each questionnaire item was ≤3, the sensitivity for diagnosing the poorly controlled asthma group (Group 1) was confirmed to be less than 80%. Therefore, the score criterion for each questionnaire item as an abnormal finding was a score of 4 or less. For the same reason, method (7) was based on the scenario in which the sum of the selected questionnaire item scores was 8 or less. For method (8), when the sum of the scores was 13 or less, the sensitivity was 99% and the specificity was 75%, and when it was 11 or less, the sensitivity and specificity were 83% and 99%, respectively. Considering this, a score of ≤12, which had high sensitivity and specificity, became the criterion for abnormal findings. The sensitivity and specificity of the survey order methods were calculated by checking the number of corresponding subjects, and the results are presented in Table 5.

Methods (7) and (8) were confirmed to have high sensitivity and specificity, depending on the survey order method. However, for method (7), the sensitivity and specificity differed depending on the choice of questionnaire. Additional analyses were conducted to determine the questionnaire item choices that were better. To help make decisions regarding the choice of questionnaire items, the correlation between the ACT total score and the sum of Q2 and Q1, as well as the correlation between the ACT total score and the sum of Q2 and Q3 were compared. The correlation between the sum and the score, after excluding each questionnaire, was also obtained and compared to avoid multicollinearity (Table 6).

## 4. Discussion

To identify a representative questionnaire for asthma control assessment in digital healthcare, this study analyzed the correlation and explanatory power of each item in ACT by using Pearson correlation values and ROC curves. Q1, Q2, Q3, and Q5 were confirmed as showing greater correlations than Q4, establishing the basis for a representative questionnaire. Q5 (How would you rate your asthma control during the past 4 weeks?) was excluded because it was relatively difficult to investigate using a digital healthcare system. When Q1, Q2, and Q3 were analyzed according to the survey order method, the sum of their scores showed the most similar results to the total ACT score.

In this study, the correlation or explanatory power of Q4, the use of emergency medication, was not higher than that of the other questions. This differed from the findings of the study that proposed the ACT, which identified Q4 as the third-most important factor [13]. However, this question has been reported to be not significant in some studies [16]. Although the results were analyzed using other asthma control assessment tools, some studies have reported that the results are not significant with or without the questionnaire confirming the use of emergency medication [17]. The reasons for this discrepancy may include differences in the number of subjects or whether the study used prospective or retrospective analysis. As mentioned in other studies, even if patients have difficulty in breathing or other asthma symptoms, they may not have used emergency medications for realistic reasons, such as the absence of medications or other reasons [17]. In a tool called Royal College of Physics 3 Questions for Asthma (RCP3Q) [18], which gathered expert opinions to produce standardized questionnaires for asthma control assessments, Q5 was not selected as a representative item for the same reason, and a clear basis for this was not provided.

Using selected representative questionnaires, this study identified the sensitivity and specificity of each survey order method and the total ACT score to diagnose asthma control status, assuming a number of ways to investigate the future use of these data in digital healthcare systems. In RCP3Q, a prospective analysis was conducted using the same three questionnaires, Q1, Q2 and Q3 using the same method compared to Method (4) of our study. It showed 96% sensitivity and 34% specificity [19]. When using the same method in our study, the results of 99% sensitivity and 53% specificity and identical tendencies were confirmed. There are limitations in RCP3Q since answering ‘yes’ to any question in those three questions can result in uncontrolled asthma status leading to too many medical interventions. This study analyzed various other combinations. One of the methods confirmed the absence of such limitations, resulting higher sensitivity and specificity (Methods 7 and 8).

The limitation of our study that the data were collected from only one center for analysis and regional characteristics were not considered, should be noted. In addition, this was a retrospective study, and the data collection period was only one year. Owing to the nature of the data, statistical limitations existed, such as multicollinearity.

The ACT is an asthma control assessment tool that patients answered while recalling their asthma control status over the past 4 weeks. This can be affected by memory errors, and quick assessments in some areas may be difficult to make when the symptoms change rapidly. A digital healthcare system is expected to solve these problems by allowing patients to immediately enter their symptoms when their asthma control status changes. However, no study has provided such evidence. The present study is meaningful in that it provides necessary evidence for the development of such systems. The results obtained from this study are thought to be a useful basis for research to determine which methods to apply when utilizing ACT questionnaires in digital healthcare systems in the future.

## 5. Conclusions

The study analyzed ACT items, which are used for assessing asthma control status, and selected representative items that could be used in digital healthcare systems in the future. The selected questions were as follows: (1) In the past 4 weeks, how much of the time did your asthma keep you from getting as much done at work, school, or home? (2) During the past 4 weeks, how often have you experienced shortness of breath? (3) During the past 4 weeks, how often did your asthma symptoms wake you up at night or earlier than usual in the morning? When using these questions to determine the research method that best reflected the results of the asthma control assessment, the method of calculating the sum of the scores of the selected questions showed the highest sensitivity and specificity. Selected representative questionnaire items in the ACT and combinations of these items could be applicable for a digital healthcare system in future.

## Figures and Tables

**Table 1:** Sex and age distribution between Asthma Control Test result groups.

		Group 1 ^a^ (*n* = 473)	Group 2 ^b^ (*n* = 1546)	Total (*n* = 2019)
Sex	Men	206	670	876
	Women	267	876	1143
Age Group	10–19	5	4	9
	20–29	26	46	72
	30–39	27	84	111
	40–49	35	147	182
	50–59	46	266	312
	60–69	125	416	541
	70–79	119	370	489
	80–89	86	197	283
	90–99	4	16	20

^a^ Group 1 stands for the group whose ACT scores are between 5~19, meaning not welled control of asthma. ^b^ Group 2 stands for the group whose ACT scores are between 20~25, meaning welled control of asthma.

**Table 2:** Correlations between scores for each questionnaire and total score of Asthma Control Test.

	Q1	Q2	Q3	Q4	Q5
Pearson Correlation Coefficient	0.818	0.822	0.778	0.393	0.739
*p* Value	<0.0001	<0.0001	<0.0001	<0.0001	<0.0001

**Table 3:** The correlation between each ACT questionnaire item and the total score after excluding the score for that item.

	Correlation of the Q1 Score with the Total Score Excluding Q1	Correlation of the Q2 Score with the Total Score Excluding Q2	Correlation of the Q3 Score with the Total Score Excluding Q3	Correlation of the Q4 Score with the Total Score Excluding Q4	Correlation of the Q5 Score with the Total Score Excluding Q5
Pearson Correlation Coefficient	0.684	0.652	0.591	0.226	0.592
*p* Value	<0.0001	<0.0001	<0.0001	<0.0001	<0.0001

**Table 4:** Asthma control test analysis of diagnostic power in groups (Group 1) where asthma symptoms are not well controlled for each questionnaire item.

	Q1	Q2	Q3	Q4	Q5
Area under the curve	0.909	0.910	0.860	0.599	0.855
*p* Value	<0.0001	<0.0001	<0.0001	<0.0001	<0.0001

**Table 5:** Sensitivity and specificity according to the survey order methods of selected representative questionnaire items for diagnosing Group 1.

Method 1	Q2 score ≤ 4	Q2 score = 5	
Group 1	558	46	
Group 2	360	1055	
Sensitivity (%)	92.0	Specificity (%)	74.0
Method 2	One of two scores (Q1, Q2) ≤ 4	Both scores = 5	
Group 1	596	8	
Group 2	535	880	
Sensitivity (%)	98.6	Specificity (%)	62.0
Method 3	Both scores (Q1, Q2) ≤ 4	One of two scores = 5	
Group 1	524	80	
Group 2	144	1024	
Sensitivity (%)	86.6	Specificity (%)	84.7
Method 4	One of three scores (Q1, Q2, Q3) ≤ 4	All three scores = 5	
Group 1	603	1	
Group 2	660	755	
Sensitivity (%)	99.0	Specificity (%)	53.0
Method 5	Two of three scores (Q1, Q2, Q3) ≤ 4		
Group 1	574	30	
Group 2	260	1155	
Sensitivity (%)	96.0	Specificity (%)	82.0
Method 6	All three scores (Q1, Q2, Q3) ≤ 4		
Group 1	425	179	
Group 2	73	1342	
Sensitivity (%)	70.0	Specificity (%)	95.0
Method 7-1	Sum of two questionnaire item scores (Q1 + Q2) ≤ 8	Higher than 9	
Group 1	577	27	
Group 2	239	1176	
Sensitivity (%)	96.0	Specificity (%)	83.0
Method 7-2	Sum of two questionnaire item scores (Q2 + Q3) ≤ 8	Higher than 9	
Group 1	568	36	
Group 2	154	1261	
Sensitivity (%)	94.0	Specificity (%)	89.0
Method 8	Sum of three questionnaire item scores (Q1 + Q2 +Q3) ≤ 12	Higher than 13	
Group 1	585	19	
Group 2	122	1293	
Sensitivity (%)	97.0	Specificity (%)	91.0

Group 1, poorly controlled asthma (*n* = 604); Group 2, well-controlled asthma (*n* = 1415).

**Table 6:** Correlation with total score based on sub-questionnaire selection in method (7).

	Correlation of the Sum of Q1 and Q2 Scores and the Total Score	Correlation of the Sum of Q1 and Q2 Scores and the Total Score Minus the Scores of Q1 and Q2	Correlation of the Sum of Q2 and Q3 Scores and the Total Score	Correlation of the Sum of Q2 and Q3 Scores and the Total Score Minus the Scores of Q2 and Q3
Pearson’s Correlation Coefficient	0.914	0.656	0.919	0.707
*p* Value	<0.0001	<0.0001	<0.0001	<0.0001

Method (7) Examine Q2 and 1 or 3 to calculate their sum (7-1: Q2 and Q1, 7-2: Q2 and Q3).

## Data Availability

The data presented in this study are available from the corresponding author on reasonable request.

## References

[1] McCracken J.L., Veeranki S.P., Ameredes B.T., Calhoun W.J. (2017). Diagnosis and management of asthma in adults: A review. JAMA.

[2] Papi A., Brightling C., Pedersen S.E., Reddel H.K. (2018). Asthma. Lancet.

[3] Ventola C.L. (2014). Mobile devices and apps for health care professionls: Uses and benefits. P T.

[4] Chongmelaxme B., Lee S., Dhippayom T., Saokaew S., Chaiyakunapruk N., Dilokthornsakul P. (2019). The effects of telemedicine on asthma control and patients’ quality of life in adults: A systematic review and meta-analysis. J. Allergy Clin. Immunol. Pract..

[5] Marcano Belisario J.S., Huckvale K., Greenfield G., Car J., Gunn L.H. (2013). Smartphone and tablet self management apps for asthma. Cochrane Database Syst. Rev..

[6] Zhao J., Zhai Y.K., Zhu W.J., Sun D.X. (2015). Effectiveness of telemedicine for controlling asthma symptoms: A systematic review and meta-analysis. Telemed J. E Health.

[7] Kim M.Y., Lee S.Y., Jo E.J., Lee S.E., Kang M.G., Song W.J., Kim S.H., Cho S.H., Min K.U., Ahn K.H. (2016). Feasibility of a smartphone application based action plan and monitoring in asthma. Asia Pac. Allergy.

[8] Anhøj J., Nielsen L. (2004). Quantitative and qualitative usage data of an Internet-based asthma monitoring tool. J. Med. Internet Res..

[9] Ostojic V., Cvoriscec B., Ostojic S.B., Reznikoff D., Stipic-Markovic A., Tudjman Z. (2005). Improving asthma control through telemedicine: A study of short-message service. Telemed J. E Health.

[10] Finkelstein J., O’Connor G., Friedmann R.H. (2001). Development and implementation of the home asthma telemonitoring (HAT) system to facilitate asthma self-care, IOS Press, Amsterdam, Netherlands. Stud. Health Technol. Inform..

[11] (2021). GINA, Global Initiative for Asthma—Global Strategy for Asthma Management and Prevention. https://ginasthma.org/.

[12] Juniper E.F., O’Byrne P.M., Guyatt G.H., Ferrie P.J., King D.R. (1999). Development and validation of a questionnaire to measure asthma control. Eur. Respir. J..

[13] Nathan R.A., Sorkness C.A., Kosinski M., Schatz M., Li J.T., Marcus P., Murray J.J., Pendergraft T.B. (2004). Development of the asthma control test: A survey for assessing asthma control. J. Allergy Clin. Immunol..

[14] Schatz M., Sorkness C.A., Li J.T., Marcus P., Murray J.J., Nathan R.A., Kosinski M., Pendergraft T.B., Jhingran P. (2006). Asthma Control Test: Reliability, validity, and responsiveness in patients not previously followed by asthma specialists. J. Allergy Clin. Immunol..

[15] Liu A.H., Zeiger R., Sorkness C., Mahr T., Ostrom N., Burgess S., Rosenzweig J.C., Manjunath R. (2007). Development and cross-sectional validation of the Childhood Asthma Control Test. J. Allergy Clin. Immunol..

[16] Gemicioğlu B., Mungan D., Bavbek S., Yıldız F., Polatlı M., Naycı S., Erkekol F.Ö., Türker H., Günen H., Çamsarı G. (2020). Validity and reliability of the assessment tool for asthma (ATA) questionnaire: The ATA study. Turk. Thorac. J..

[17] Juniper E.F., Svensson K., Mörk A.C., Ståhl E. (2005). Measurement properties and interpretation of three shortened versions of the asthma control questionnaire. Respir. Med..

[18] Georgiou A., Pearson M. (2002). Measuring outcomes with tools of proven feasibility and utility: The example of a patient-focused asthma measure. J. Eval. Clin. Pract..

[19] Pinnock H., Burton C., Campbell S., Gruffydd-Jones K., Hannon K., Hoskins G., Lester H., Price D. (2012). Clinical implications of the Royal College of Physicians three questions in routine asthma care: A real-life validation study. Prim. Care Respir. J..

